# Lao PDR as an emergency preparedness model for developing countries: Lessons learned from the COVID-19 pandemic

**DOI:** 10.7189/jogh.13.03040

**Published:** 2023-08-18

**Authors:** Randa Elsheikh, Abdelrahman M Makram, Sajog Kansakar, Khamsamay Xaylovong, Hirotsugu Aiga, Nguyen Tien Huy

**Affiliations:** 1 Deanery of Biomedical Sciences, Edinburgh Medical School, University of Edinburgh, Edinburgh, UK; 2 Online Research Club, Nagasaki, Japan; 3 School of Public Health, Imperial College London, London, UK; 4 Department of Internal Medicine, Manipal College of Medical Sciences, Pokhara, Nepal; 5 Provincial Health Department, DCDC, Savannakhet Province, Lao PDR; 6 School of Tropical Medicine and Global Health (TMGH), Nagasaki University, Nagasaki Japan

Despite sharing a border with China, the Lao People’s Democratic Republic (Lao PDR) announced its first COVID-19 case only on 24 March 2020, becoming the last country in Southeast Asia to report a confirmed case of the virus [1]. By the end of 2020, the country reported 40 confirmed COVID-19 cases and zero deaths, making it one of few that had succeeded in preventing the importation of the virus. However, after reporting only several cases in early 2021, a rapid surge occurred at the end of April 2021, rising to around two thousand cases and three deaths [2]. This raised questions regarding the causes underlying the sudden increase in cases, highlighting the future implications this had and the necessity of revising the responses that had until then protected Lao PDR.

Following the identification of nine cases by the end of March 2020 and the rapidly spreading outbreak in neighbouring Thailand, the Laotian government established a lockdown on 30 March 2020, banning international travel (except for key personnel) and mandating testing and quarantine of incoming visitors and migrant workers [3]. Lao PDR also benefited from being geographically surrounded by countries that have been relatively successful in controlling the spread of COVID-19 (i.e. Cambodia, China, Myanmar, and Thailand), making importation of virus carriers into the landlocked country less likely [1]. Additionally, Lao DPR is one of the most rural countries in Southeast Asia, with 64% of the country’s population residing in rural areas [4]. While this makes transmission less likely, it may also place Lao PDR at risk of emerging coronaviruses [1,5]. Furthermore, rural areas may have higher mortality rates due to inaccessibility to healthcare facilities [6]. The country had reported zero deaths up to May 2021 [7], over a year into the pandemic, possibly because most infections occurred among young individuals aged 15-34 years old. A combination of these factors may explain how Lao PDR has been able to manage the COVID-19 pandemic for so long.

Restrictions were relaxed within the next two months (from June to July 2020) and certain businesses, domestic travel, and schools began opening again. By June 2020, the government declared victory against COVID-19 following the discharge of all COVID-19 cases from hospitals, reporting no new cases for two months. Simultaneously, all individuals entering the country continued to be monitored with a 14-day mandatory quarantine, and in July 2020, over four thousand individuals were being monitored in isolation across the country [3]. Despite only a few confirmed cases, COVID-19 prevention measures continued through the end of 2020, such as restriction of mass gatherings, closure of entertainment venues, suspension of flights from countries with local transmission, suspension of issuance of tourist visas, closure of border checkpoints, extra monitoring of those entering Laos, and a requirement for those entering Laos to wear a medical device for tracking purposes. Certain regions of the country entered immediate lockdowns upon the reports of imported cases. Thus, despite only a sporadic number of cases, the Lao authorities remained vigilant.

However, in 2021, Virachith et al. [8] investigated the possibility of the disease burden in Lao PDR being much higher, assuming that the virus spread silently among asymptomatic young individuals. They measured the seroprevalence of SARS-CoV-2 antibodies in a sample of 3173 individuals, in what represented the largest COVID-19 seroprevalence study in Southeast Asia. Their results showed that only 0.1% of Laos’ population had antibodies to SARS-CoV-2 nucleoprotein and spike protein, which confirms the good virus control achieved by the country during the first year of the pandemic [8], but also highlights a lack of immunity against the virus [1].

**Figure 1:**
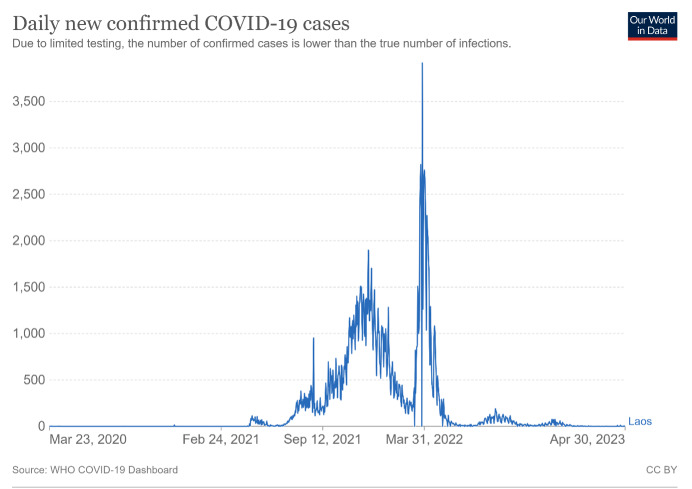
The incidence of COVID-19 cases in Lao PDR from 23 March 2020 to 14 June 2023. Adopted from Our World in Data [9], free to use under Creative Commons BY 4.0 license.

After keeping the daily incidence below fifty for a long period (**Figure 1**), the number of infections spiked to thousands and the number of deaths to a few hundred. The new clusters were linked to casinos in the Golden Triangle border zone with Myanmar and Thailand, migrant workers who have returned from Thailand, and the Lao New Year celebrations in mid-April 2021. This sharp rise in the number of cases challenged the public health authorities in coordinating case investigation, contact tracing, and case management, while the health care system was overwhelmed by the rapidly rising number of hospitalisations. These issues were exacerbated by the country’s limited resources. Nevertheless, the number of cases dwindled; on 31 May 2021, the Lao authorities reported no new community cases of COVID-19 [9].

However, the emergence of new COVID-19 variants since January 2022 led to new waves of infections in the country. The situation especially deteriorated in March when the country registered 24 031 cases and 15 deaths in a few days, bringing the total number of deaths to 757 since the onset of the pandemic. In May 2022, however, the number of cases plummeted, and COVID-19 restrictions were consequently eased [10].

These recurring outbreaks provide the public health community with an important lesson that no system is completely secure, and that contingency planning and emergency preparedness need to be implemented for any upcoming infectious disease outbreak or pandemic. Nevertheless, Lao authorities successfully stepped-up vaccination efforts, becoming the sixth country in Southeast Asia to reach the 70% landmark of vaccinating their population by mid-2022 [11]. In one year, full vaccination rates went from 3.2% to 69.78%. Meanwhile, 78.9% of the population received at least one dose of the vaccine, which is a major improvement compared to 8.9% in May 2021 [12,13]. As of May 2022, 20.5% of the Laotian population had received one booster dose and 1.4% a second booster dose [14].

The successful implementation of COVID-19 vaccination plans was done after the Laotian government realised their shortcomings, manifested in the shortage of healthcare professionals, the unavailability of proper isolating facilities for infected patients, the inaccessibility of care and vaccination in remote areas, and the slow uptake of vaccines in the general population during the 2021 outbreaks [15]. As the government had already been working on achieving Universal Health Coverage by 2025, it easily managed to redistribute the workforce to key areas and provide training to individuals lacking knowledge on dealing with infectious diseases and respiratory distress. The government also made sure that specialists are well-equipped with the latest up-to-date clinical knowledge and skills [16]. The Laotian government worked on renovating roads to remote areas, expanding isolation facilities, and creating the Lao PDR COVID-19 response project to enhance public health preparedness for the pandemic [17]. Lastly, with some help from UNICEF Lao PDR, the authorities were able to distribute personal protective equipment, oxygen therapy equipment, and other medical supplies to hospitals [18].

Despite being a low-income country and one of the poorest in Southeast Asia, Lao PDR has not only been able to prevent significant COVID-19 transmission, but also successfully dealt with the new waves of infections. The 2022 Lao PDR way of implementing public health responses may serve as a model for other resource-constrained LMICs.
